# Net Loss? Agrochemicals and Insecticide Resistance in the Fight against Malaria

**DOI:** 10.1289/ehp.125-A50

**Published:** 2017-03-01

**Authors:** Winifred A. Bird

**Affiliations:** Winifred A. Bird is a freelance journalist living in northern Illinois. Her work has appeared in *Science*, *Yale Environment 360*, *Sierra*, and other publications.

*To report this story, Winifred Bird traveled to the laboratories and cotton fields of western Burkina Faso, a small country in West Africa. She is indebted to her translator Dramane Soh for navigating between French, Jula, and the multiple local languages spoken in the small farm towns they visited.*

In the middle of the night, Eugene Somda’s 9-month-old son began to convulse uncontrollably. Moments later, burning with fever, he lost consciousness. Somda was terrified. A 55-year-old farmer and father of seven in the Dano district of southwestern Burkina Faso, he knew immediately what was wrong: His youngest child had a severe case of malaria. The *Plasmodium* parasites that cause malaria were rapidly multiplying out of control, shattering his red blood cells, releasing toxins, and interfering with his blood flow. The farmer and his wife rushed the baby down three kilometers of bumpy red dirt roads to a health clinic. The child was admitted and, to his parents’ great relief, slowly recovered over the next four days.

**Figure:**
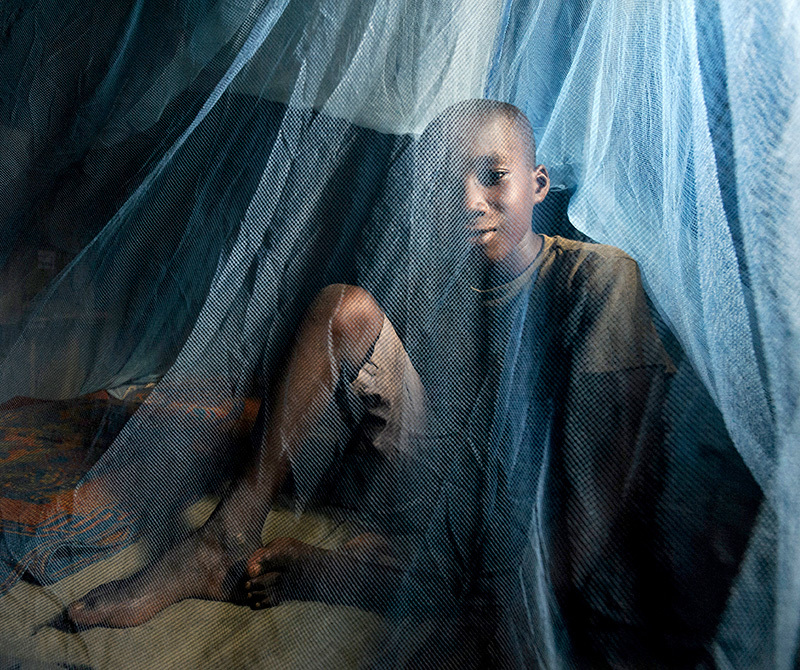
A combination of indoor spraying and use of insecticide-treated bed nets has slashed cases of malaria, but now researchers are reporting widespread resistance of mosquitoes to the chemicals used to kill them. Agricultural pesticides appear to be playing a role in fostering this resistance. © Nyani Quarmyne/Panos Pictures

Were his son a few years older, Somda likely wouldn’t have worried much. Malaria is so common in this part of the country that most children contract the disease at least once each rainy season.^1^ With repeated exposure comes natural immunity and, typically, freedom from debilitating symptoms; unless the case is severe, parents treat their children at home and continue with daily life as best they can.^2^ But in children under age 5, who have not yet built up immunity, the disease can be deadly. In 2015 it killed a reported 4,005 young Burkinabé children,^3^ more than any other cause of death. Across Africa, where the vast majority of malaria cases occur, it killed an estimated 292,000 children under age 5.^4^

In one sense, Somda’s story belongs to a global narrative of triumph over a long-neglected disease. Thanks to a surge of international funding and attention,^5^ the annual number of malaria cases worldwide has fallen by 59 million over the past 15 years, and the number of deaths has been cut approximately in half; in 17 countries the disease has vanished entirely.^4^

In another sense, however, his story is foreboding. That is because one important tool in the fight against malaria is beginning to fail. Recent research shows that throughout sub-Saharan Africa and beyond, mosquitoes are becoming resistant to the insecticides used to control the *Anopheles* mosquitoes that spread the disease.^6^ ^,^ ^7^

## Growing Resistance

In Africa, a combination of better medicines and the widespread use of insecticides to kill mosquitoes has led to the decline in malaria cases and deaths.^4^ Millions of people in places like Dano are living longer, healthier lives because of this public health campaign. They are also more prosperous when they are malaria free, because the disease keeps kids home from school, prevents adults from working, and forces poor families to spend money on health care.^8^

**Figure:**
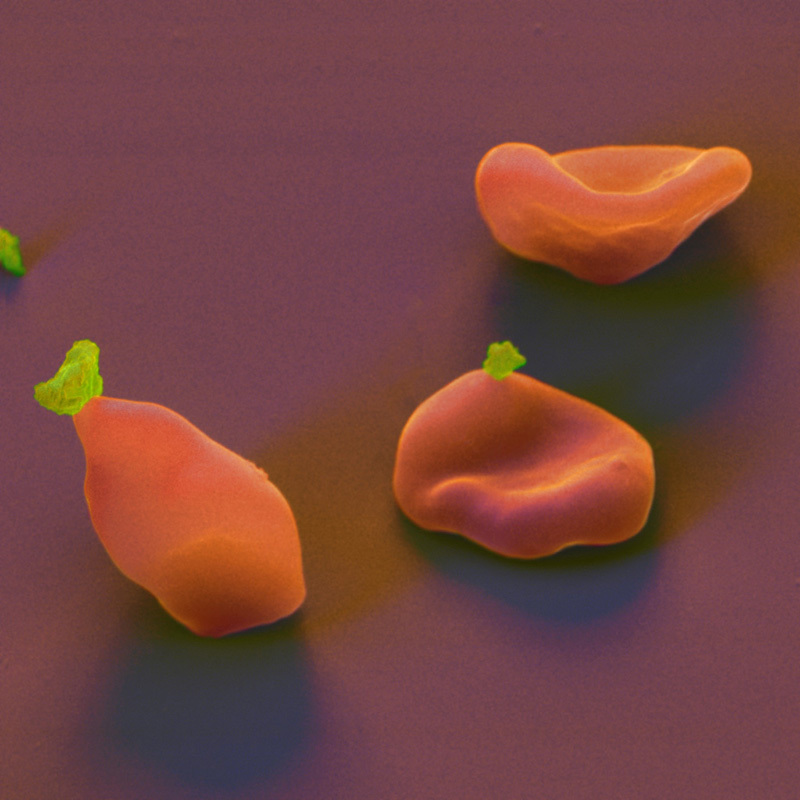
Plasmodium merozoites (or daughter parasites, shown here in green) invade red blood cells by attaching themselves and injecting enzymes and lipids that weaken the cell membrane. The parasite then enters the cell and reproduces. Within 48 hours the cell bursts, releasing more merozoites to infect additional red cells. © Eye of Science/Science Source

However, in recent years more than three dozen countries have reported confirmed resistance of *Anopheles* mosquitoes to pyrethroids,^9^ the only class of insecticide currently used on bed nets (which are a key element of malaria prevention) and, until recently, the primary class used in indoor residual sprays.^10^ Even more have reported resistance to carbamates, organophosphates, or organochlorines, the other three classes used in sprays.^9^ In a few places, mosquitoes are resistant to all four classes of insecticides, leaving no chemical option to control them whatsoever.^11^

Farmers like Somda may unwittingly be part of the reason that is happening. A smallholder in the heart of Burkina Faso’s cotton belt, he grows a hectare of that crop, which is second only to gold in economic importance for the country.^12^ Despite occupying just 5% of farmland, cotton accounts for 90% of the pesticides applied in Burkina Faso.^13^ Six times each summer, as Somda’s plants grow from tiny sprouts to waist-high stalks exploding with puffs of white, he straps on a plastic backpack sprayer and douses his fields with pesticides. His neighbors do the same. Some of the compounds they use are pyrethroids, the same family of chemicals that lace the billowy white nets strung above their beds. When it rains, these pesticides trickle off their fields and into the puddles where mosquitoes lay their eggs.

Scientists studying the region believe that the resulting exposure of generation after generation of larvae to agrochemicals is a key reason why treated bed nets no longer kill adult mosquitoes effectively. “We found the same pyrethroids in the breeding sites as in the fields, and we concluded that these small doses in the water are the source of resistance selection in this area,” says Aristide Hien, an entomologist who recently compared pesticide contamination and resistance levels in sections of Dano growing conventional versus organic cotton. His results have been submitted for publication.

Hien belongs to a team of scientists at the Institute de Recherche en Sciences de la Santé (IRSS), a government research center not far from Dano in the city of Bobo-Dioulasso. For the past 15 years this team has been studying the relationship between insecticide resistance and all four classes of chemicals currently used in vector control.^14^ Together with researchers in Tanzania, Benin, Cameroon, and several other countries, they are building a body of evidence that suggests the heavy use of agrochemicals—especially on cotton and vegetable farms—is one factor in growing resistance.^14^ ^,^ ^15^

The extent and significance of agriculture’s impact remain controversial. The evidence is largely circumstantial, and other factors—particularly the intensive use of insecticides for malaria control itself—unquestionably play a role in selecting for resistance. It is also unclear whether resistance is actually undermining malaria control programs.^16^ Pesticide resistance has been convincingly linked to an increase in illnesses in only one instance, when South Africa’s switch from DDT to pyrethroids for vector control was followed by a quadrupling of malaria cases between 1996 and 2000.^17^ However, a review of 75 malaria resurgence events since 1930 concluded that 14 of them may have been caused by insecticide resistance.^18^ According to many who study the problem, the evidence that does exist calls for much stronger action by governments and international bodies than is currently being taken.

**Figure:**
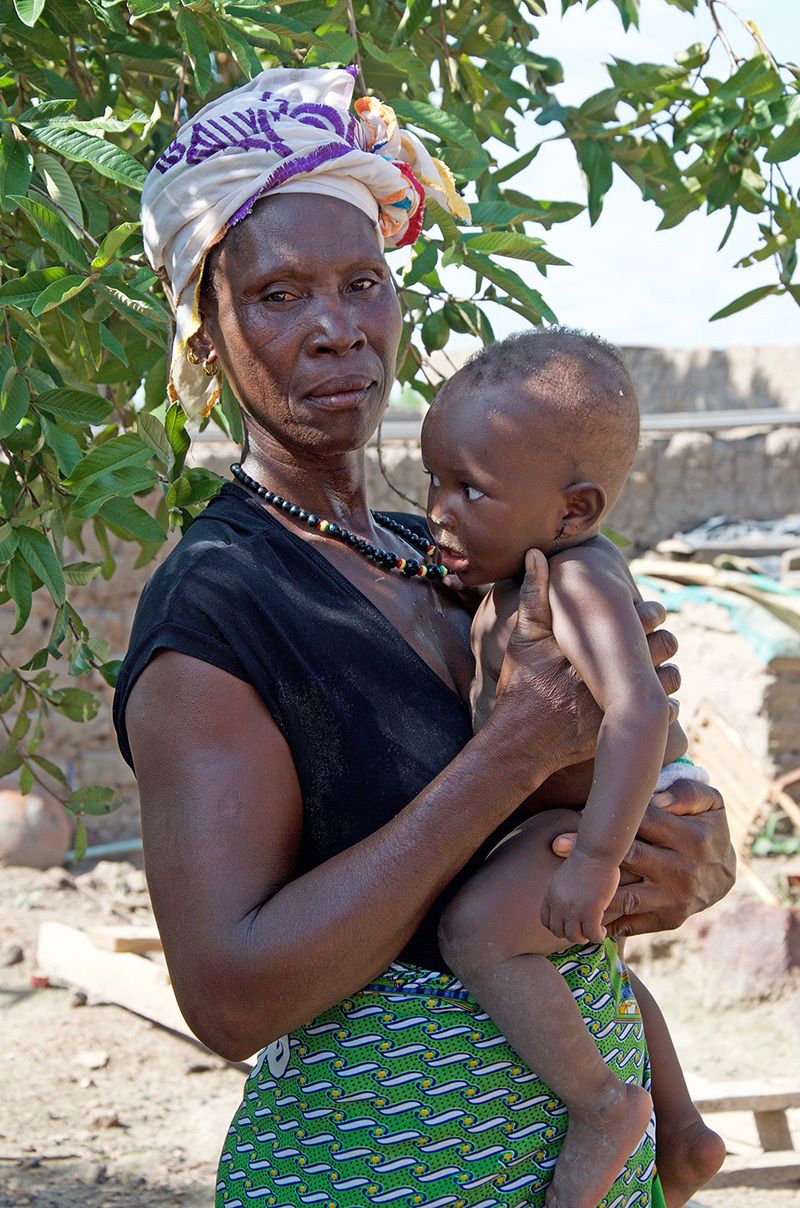
Salmata Ouedraogo, a farmer in the village of Bama, had malaria last year, although her baby daughter did not. Older children and adults acquire immunity with repeated infections and often show no symptoms. But in children under 5 who have not yet built up immunity, the disease can be deadly. © Winifred Bird

“Resistance is not only a problem of health but also of agricultural producers,” says Roch Dabiré, research director at IRSS and a medical entomologist. “The GPIRM clearly addresses the problem of agriculture,” he says, referring to the World Health Organization (WHO) Global Plan for Insecticide Resistance Management. “But what are they doing to solve the problem?” Dabiré believes coordination between the agricultural and public health sectors, better pesticide management, and new chemical and nonchemical tools to fight malaria are all urgently needed.

The stakes of inaction are high. The use of public health interventions is estimated to have averted 663 million cases in Africa between 2000 and 2015, of which approximately 517 million were attributed to the use of insecticide-treated bed nets and indoor sprays.^19^ No other currently available tool has the same power to combat the disease in poor, highly endemic countries like Burkina Faso. Yet while new chemicals are being developed to replace those that have begun to fail, history and biology suggest that mosquitoes will become resistant to these, too, unless they are used with extreme care. Vector biologist Jo Lines sees in that cycle of resistance the potential for a public health disaster.

“Is this an arms race we can win? If it’s not, what do we do then?” asks Lines, a malaria control expert at the London School of Hygiene and Tropical Medicine, who led the development of the GPIRM. “Imagine you’ve got an entire continent halfway to elimination [of malaria], so half the people have been living for five or ten years without malaria, and they’ve lost their immunity. We know that unless we keep up really thorough mosquito control there will be catastrophic epidemics and huge loss of lives that drugs can’t prevent. It’s a dangerous situation.”

## The Agriculture Connection

The first reports suggesting a link between agriculture and resistance to the insecticides used against malaria vectors date to the mid twentieth century.^14^ The WHO had just launched its first global campaign to eradicate malaria, and both DDT and dieldrin were being sprayed inside houses across Africa, Asia, the Americas, and Europe.^20^ Both chemicals had also been introduced globally as agricultural pesticides. When resistance very quickly became a problem in the malaria eradication campaign, it seemed logical that agriculture must be playing a role. That, at least, was the conclusion of a 1962 study by the WHO, which in some areas attributed vector resistance “mainly, and in some places entirely, to the use of agricultural insecticides.”^21^ The paper singled out in particular the “unrestrained use” of dieldrin on cotton.

Almost 20 years later, when Lines arrived in Sudan on his first field assignment, he came across the same claim—although this time the suspected culprits were organophosphates used on vast industrial farms along the Nile. By then the WHO’s first eradication campaign had fallen victim to politics, insecticide-resistant mosquitoes, and drug-resistant parasites, and a resurgence of malaria was under way in many places where the campaign had been working.^17^ Nevertheless, in Sudan houses were still being sprayed, and mosquito resistance to the organophosphate malathion had emerged after only four rounds.^22^

**Figure:**
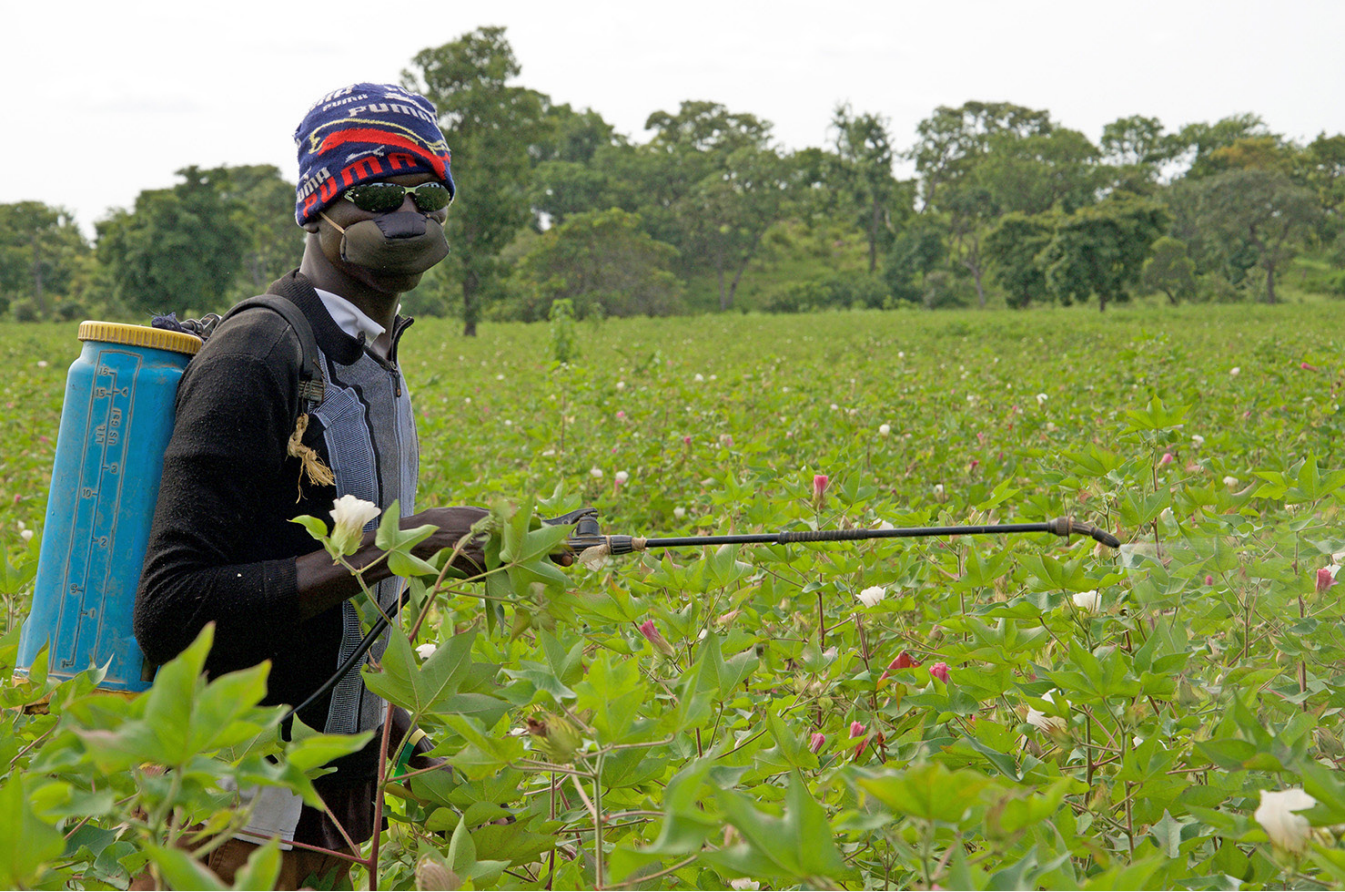
Burkina Faso is famous for its luxurious long-staple cotton, which is second only to gold in economic importance for the country. Despite occupying just 5% of farmland, the crop accounts for 90% of the pesticides applied in Burkina Faso. © Winifred Bird

**Figure:**
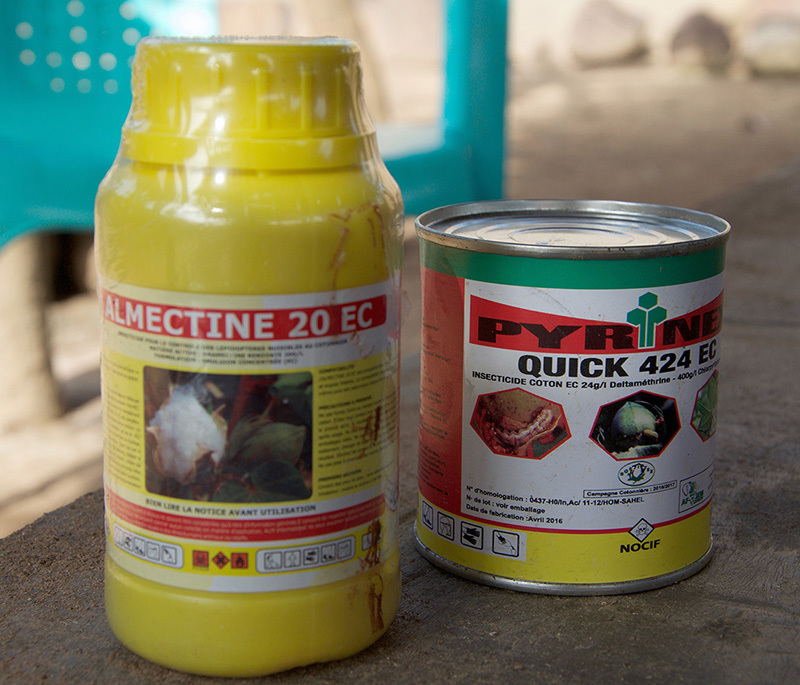
In this region, pesticides are often sold on the black market, used haphazardly, and disposed of incorrectly. Furthermore, farmers rely heavily on the specific chemicals that most need to be controlled, such as deltamethrin, a key insecticide in fighting mosquitoes. © Winifred Bird

“Everybody was blaming agriculture,” Lines recalls. But he himself was doubtful. Was the problem really agriculture, or was it the aggressive public health campaign itself? Together with a colleague, he investigated the question and found that resistance aligned geographically and temporally with indoor residual spraying, not agricultural spraying. In 1988 he published a paper comparing the situation in Sudan to similar ones in El Salvador and Sri Lanka.^22^ He concluded that the impact of agricultural spraying varied widely depending on the behavior of the specific pest involved—where it bred, slept, and ate, and thus where it might be exposed to agrochemicals. “People go, ‘Oh, it’s obvious,’ but no, it’s not obvious,” he says.

The problem received little attention for the next decade. Then in 2002, just as the WHO was ramping up a new campaign to eliminate malaria, a young Burkinabé scientist named Abdoulaye Diabaté led a study that sparked interest in the topic among a new generation of African researchers. His focus was the pyrethroid family of insecticides. Introduced into agriculture in the 1970s and vector control in the late 1980s,^23^ pyrethroids were cheap, effective, and relatively safe for birds and mammals. They could also be incorporated into bed nets, which meant that instead of visiting villages once or twice a year to spray houses, public health officials could simply distribute nets that worked for up to three years.^24^ The nets both created a physical barrier around the person sleeping inside and killed any mosquito that landed on them, thereby lowering the overall number of vectors and protecting the entire community. Bed nets soon became the centerpiece of vector control in Africa.

Predictably, reports of resistance quickly emerged. In Burkina Faso, Diabaté (who is now on the staff of IRSS) was tracking the distribution of resistant mosquitoes,^25^ and he discovered a remarkable geographical correlation with the country’s cotton belt. He also found that mosquitoes collected during the rainy season, when farmers apply pyrethroids to their fields, were more likely than those collected at other times to show resistance in the lab.^26^ It wasn’t proof of cause—he had not ruled out factors like public health campaigns or existing resistance to DDT, which confers some cross-resistance to pyrethroids because they both target the same nerve cell proteins.^27^ But over the next decade and a half, he and other scientists continued to gather similar evidence.^14^ ^,^ ^15^ ^,^ ^28^

“Whenever we screen for resistance, it’s always high where insecticides are used intensively [in agriculture],” says Hyacinthe Toé, an entomologist at Burkina Faso’s National Center for Research and Training on Malaria. Recently, Toé tracked increases in the strength of resistance to the pyrethroid deltamethrin in mosquitoes collected from a rice- and cotton-growing area just north of Bobo-Dioulasso.^29^ ^,^ ^30^ First, he exposed groups of the mosquitoes to the chemical and measured how long it took for half of them to die; he found that the time increased tenfold in a single year. Next, he determined the concentration of insecticide needed to kill half the mosquitoes within 1 hour. It turned out the mixture had to be over 1,000 times as strong as what would kill a susceptible laboratory strain, making the wild mosquitoes the most resistant ever reported.

**Figure:**
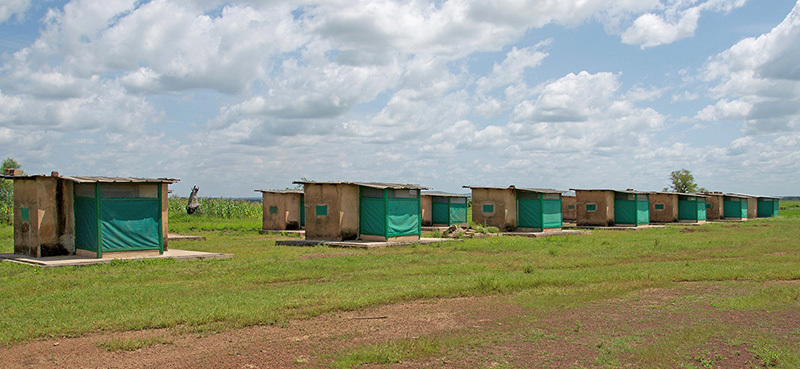
Researchers from IRSS use these huts in the village of Bama to investigate the effectiveness of indoor residual spraying and bed nets against mosquitoes. The huts are designed to resemble homes in the area, and villagers are paid to sleep in them to attract mosquitoes. © Winifred Bird

**Figure:**
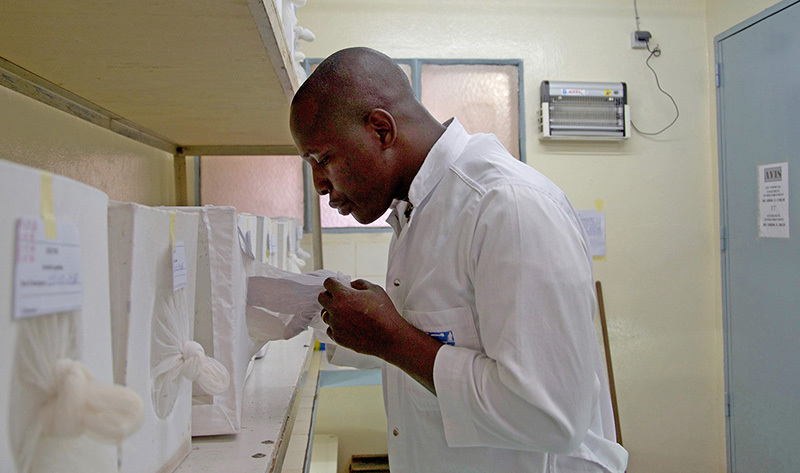
In a laboratory at IRSS, researcher Bayili Koama is raising a strain of susceptible mosquitoes. Mosquito eggs are hatched in bowls of water, and the larvae are transferred to net-covered boxes. The mosquitoes are used in various tests comparing their resistance levels to those of wild strains. © Winifred Bird

“It’s very common to find mosquitoes resting on [bed] nets. The insecticides are nothing for them,” Toé says. However, there is widespread debate over whether resistance necessarily reduces the efficacy of bed net campaigns.^31^ Intact nets still block mosquitoes, and some research suggests that even if the chemicals on nets do not kill mosquitoes immediately, they may shorten their lives.^16^

Toé suspects that skyrocketing resistance is due to “double selection” from agriculture and vector control. He explains that larvae are initially exposed to pyrethroid runoff from fields, then adult mosquitoes encounter the pyrethroid-treated bed nets hung in nearly every house. The intensity of exposure in these two settings is very different, however: low in agriculture, where runoff is diluted with rainwater and mosquitoes are not directly targeted, but high in public health, where chemicals are applied full-strength.

Hilary Ranson, a medical entomologist at the Liverpool School of Tropical Medicine who works closely with Toé and other researchers in Burkina Faso, says that difference may be crucial on a genetic level. According to multiple experts interviewed for this story, resistance develops because mutations constantly occur in any population, and some of these mutations allow individuals to survive exposures to chemicals that kill others of their kind. In mosquitoes, one common mutation prevents insecticide molecules from binding to their target sites in the nervous system. Another mutation improves the mosquito’s ability to metabolize insecticides, so the chemicals never reach the target site in the first place. But mechanisms such as these are not very powerful on their own. That means that if a population is exposed to high concentrations of insecticide from the start, individuals usually cannot survive and reproduce, even if they have the mutation. Only those with unusually powerful mutations or multiple complementary mutations will survive.

On the other hand, if a population is continuously exposed to low levels of insecticide, genes for a range of weak resistance mechanisms become common within the population. If that population is then exposed to high levels of insecticide, individuals will be much more likely to have one of these genes plus a new mutation, which together allow them to survive the intensive exposure. Although it is difficult to prove this is happening in the field, Ranson, Lines, and others believe agricultural exposures may in this way be priming populations of mosquitoes for rapid explosions of resistance once intensive malaria prevention efforts begin.

Theresia Nkya, a medical entomologist in Tanzania who has published research linking resistance to agriculture,^32^ ^,^ ^33^ says exposing larvae to low concentrations of agricultural chemicals also makes them more tolerant to those chemicals as adults. On a genetic level, Nkya says, that happens because low-level exposure triggers the overexpression of the genes that allow mosquitoes to metabolize insecticides.

None of this means that resistance spreads only when farmers use the same chemicals as public health campaigns; on the contrary, the position of the WHO and many scientists is that the primary selection pressure is coming from bed nets and indoor spraying.^17^ But if Ranson, Toé, Dabiré, and dozens of other scientists are correct, unchecked use of agrochemicals is both exacerbating the problem and hindering attempts to solve it.

## Uncoordinated Efforts

In the city of Bobo-Dioulasso, not more than a 15-minute drive from the cool tiled hallways where Dabiré and his colleagues are untangling the environmental roots of resistance, is the headquarters of the National Union of Cotton Growers of Burkina. The union represents over 350,000 farm owners, who with their families and employees comprise most of the cotton growers in Burkina Faso, says Miriam Onadia, public relations manager for the organization. Together with the country’s three large cotton companies, the union has an extremely high degree of influence over what pesticides those farmers use and how they are applied.^34^ Conceivably, it could play some role in resolving the problems that Dabiré’s team is studying. Yet neither the union’s vice secretary general, Niekiebo Kambi, nor its two press secretaries had ever heard that agrochemical use might be impacting malaria transmission. Instead, Kambi says, decisions about pesticide use are based on economic considerations.

The situation is mirrored on the national and international levels. Everyone from the WHO on down seems to agree that it is essential for the public health and agricultural sectors to coordinate their pesticide use but that in reality they almost never do. Maintaining or restoring the efficacy of a particular chemical requires that selection pressure be lifted so that susceptible mosquitoes can multiply. Typically, this is done by rotating classes of chemicals, mixing them together, or applying them in mosaic patterns so that different classes cover different patches of a particular region.^17^ But these measures are meaningless if control is not coordinated between sectors.

“You could introduce the most rational and well-resourced resistance management program for malaria, but if there are farmers who are then applying their insecticides to their fields [unfettered] it might all be undone,” says Ranson. “It’s very important for the two sectors to work together, but there’s not much incentive for them to work together at the moment.” For one thing, the benefits of using pesticides are immediately obvious to farmers, whereas the benefits of modulating use for the sake of public health are generally harder to demonstrate.

Many scientists are trying to improve the situation through farmer education and other measures. In Burkina Faso, Dabiré is preparing to present research demonstrating the agriculture–resistance link to the cotton producer’s union, a major cotton company, and the Ministry of Agriculture. In Tanzania, Nkya is lobbying policy makers to set up an intersectoral committee that could both encourage farmers to use chemicals correctly and gather information from them to inform public health decisions about vector control. In Sudan, a similar committee has functioned with some success since 2006, according to Hmooda Kafy, head of the Vector Management Unit in the Sudanese Federal Ministry of Health.

**Figure:**
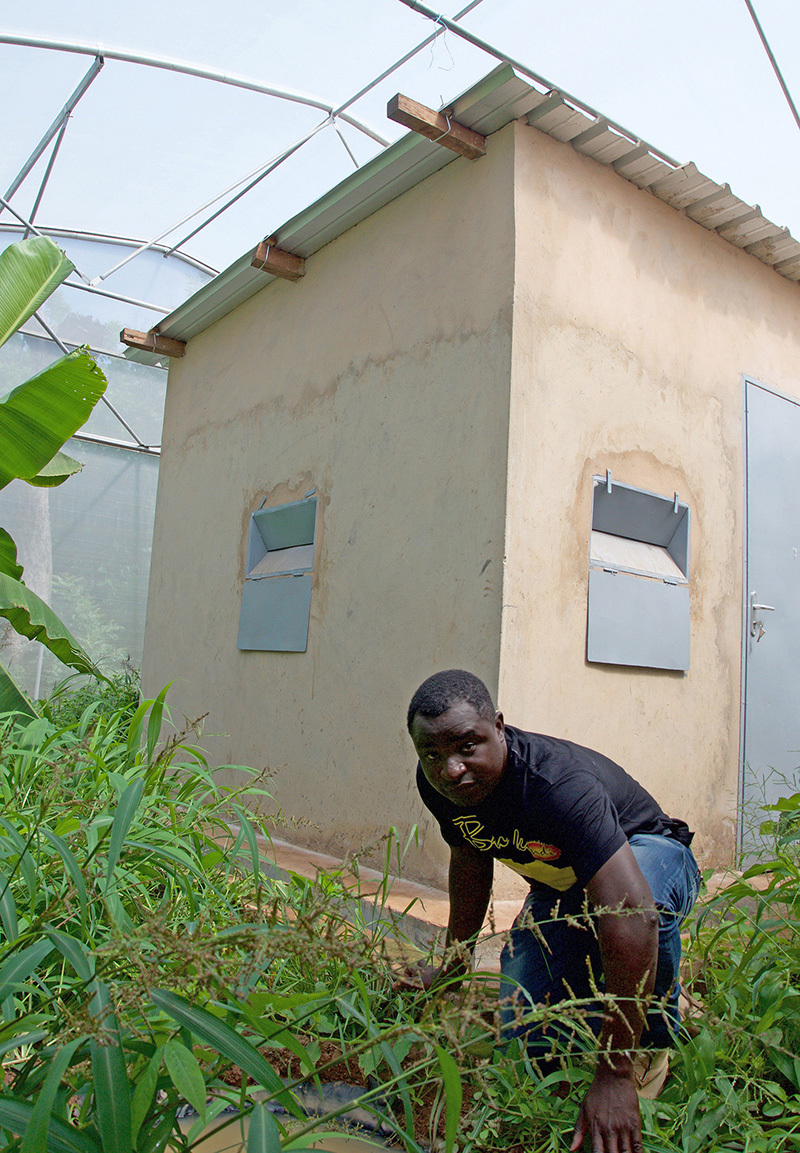
Researchers are looking for other ways to effectively fight malaria without the use of insecticides. In a replica of a village built inside a large tent-like structure, Etienne Bilgo is preparing to release a mosquito-killing fungus. © Winifred Bird

These efforts face many challenges. In sub-Saharan Africa, pesticides are often sold on the black market, used haphazardly, and disposed of incorrectly due to lack of education and guidelines.^35^ ^,^ ^36^ Furthermore, farmers rely heavily on the same chemicals that need to be controlled, and while organic production of cotton and other cash crops is increasing, it still represents a miniscule portion of the whole.^37^

In Burkina Faso, the introduction of genetically modified cotton in 2008 did lead to a large drop in pesticide use for a period of several years—by 2015 pest-resistant Bt cotton made up 70% of hectares growing the crop, and spraying on these fields was reduced from six times per season to two. However, in the process of inserting the Bt genes that make the cotton pest resistant, the staple fiber length was reduced. This was disastrous, because Burkina Faso had been known for its top-quality long-staple cotton. The program was discontinued in 2016, and pesticide use bounced back to previous levels.^38^

The current lack of alternative malaria-control pesticides also makes resistance management difficult. Since the only pesticides currently approved for use on bed nets all happen to be pyrethroids, countries have nothing to switch to once resistance becomes a problem. A few nets using combinations of alternative chemicals are in advanced stages of development.^39^ ^,^ ^40^ However, these typically still depend on pyrethroids to some extent. In the case of indoor sprays, alternatives to pyrethroids do exist, but they are more expensive,^10^ and in some places mosquitoes are resistant to them as well. For these reasons—in addition to a basic lack of resources—few African countries have followed the recommendations set forth by the WHO in its 2012 resistance management plan.^41^

But by now, says Ranson, pyrethroids may be a lost cause anyway. “We’ve got such high levels of pyrethroid resistance in malaria vectors that even if all sectors did everything they could to reduce the selection pressure on pyrethroids, I doubt we’d see a restoration of susceptibility,” she explains. So she and many others are looking ahead to the next generation of vector control chemicals, and to what they can do to ensure the same problems do not repeat themselves in the future.

One potential way to eliminate selection pressure from agriculture is to prevent new classes of vector control chemicals from being used on farms altogether. This is appealing in theory but very hard to accomplish. That is because the overlap between the chemicals used in vector control and those used in agriculture is not a coincidence; it is instead the direct consequence of economic realities in the chemical industry.^24^

## Economic Factors in Resistance

Like agricultural pesticides, vector control insecticides are developed mostly in the private sector by large diversified chemical companies. The cost of developing, testing, and registering a new active ingredient for vector control is quite high—over $175 million by one estimate.^42^ That’s why companies typically spin off vector control products from existing agricultural products and why they sell products using the same active ingredients in both markets. Of course, the final products themselves are not identical; as Lines explains, agricultural insecticides may contain solvents and other ingredients that would not be safe in a public health product.

The cost factor is also why staff in vector control divisions cannot always take actions that make sense from a resistance management perspective. Vector control insecticides are vastly less profitable than agrochemicals, with the former bringing in about $1 billion per year globally compared with $50 billion for the latter.^24^ “We as the ‘little stepbrother’ cannot say to our big brother, ‘Okay, take this active ingredient from the [agriculture] market because we are now finding that it has good efficacy against vectors,’” says Gerhard Hesse, the recently retired global partnering manager for vector control at Bayer, which is a leading producer of agrochemicals.

Some vector control chemicals end up being taken off the market for economic reasons. According to Dan Strickman, a senior program officer in vector control at the Bill and Melinda Gates Foundation, that is what happened with resmethrin and allethrin, two pyrethroids used in mosquito control. “There have been several chemicals like that,” he says. “It’s not that they were not useful; they were extremely useful for vector control. But there just weren’t enough sales to support the studies required to ensure their safety.”

The likelihood of companies developing new classes of chemicals solely for vector control is similarly low. But recently, an initiative has emerged to help them do so. It is called the Innovative Vector Control Consortium, or IVCC, and some experts say it is the world’s best chance at a chemical solution to insecticide resistance.

IVCC is a public–private partnership established in 2005 by Janet Hemingway, the director of the Liverpool School of Tropical Medicine, to develop new vector control tools. An eminent vector control biologist, Hemingway foresaw the current wave of pyrethroid resistance before many others.^43^ She also recognized that industry was not solving the problem on its own. New products were not coming down the pipeline quickly enough, and they were not being managed correctly once they were on the market. So she decided to bring together chemical companies, academics, and funders in a product development partnership similar to those that exist for pharmaceutical drugs.^44^

Armed with an initial grant of $50 million from the Bill and Melinda Gates Foundation and funding from the UK’s Department for International Development, the U.S. Agency for International Development, and the Swiss Agency for Development and Cooperation, IVCC convinced industry giants like Bayer, Sygenta, Sumitomo, and BASF to comb their chemical libraries for molecules that might be developed into insecticides to fight malaria vectors. These companies have so far narrowed a field of 4.5 million chemical candidates to just nine; the ultimate goal is to develop three new active ingredients that can be brought to market by 2022.^44^

The purpose of this effort is to develop novel compounds that would kill mosquitoes in entirely new ways, so in theory they likely would be unaffected by the resistance mechanisms that already exist in wild populations. The hope is that their efficacy could be maintained until malaria is eradicated—provided they were deployed with extreme care in both public health and agriculture.

Nick Hamon, IVCC’s current CEO, says the consortium is unlikely to demand that new active ingredients be kept out of agriculture altogether, in part for financial reasons (the narrower the potential market for new chemicals, the more IVCC must contribute to companies to help develop them). Instead, he is negotiating agreements that would limit where and how companies are allowed to market them in agriculture. “We will encourage these companies to stay out of malaria-endemic countries for a certain number of years,” he says.

John Lucas, a senior business development manager at Sumitomo, is sympathetic to that demand. “I think [IVCC has] every right to say they want to try to preserve the efficacy of these chemistries, so let’s make sure they’re not used heavily in ag and other areas,” he says.

Despite such accommodations, Hamon remains keenly aware of the economic realities driving the corporations he partners with. “I worry every day about these companies saying ‘we’ve had enough,’” he says. He estimates that in the past 20 years, the number of companies capable of doing basic research on novel vector control insecticides has fallen from over 15 to only five or six, as companies have bought up one another, reducing the options IVCC has for partnering. “They’ve got bigger fish to fry than us,” he says. “And so we are very dependent on their goodwill.”

The issue may be moot; according to Hamon, most of the nine chemicals IVCC is currently examining in detail do not look very promising for use against agricultural pests. “It’s great because now you’ve got some tools that are ‘owned’ by public health, but on the other hand the markets are very, very small, so we have to spend a lot more time finding people who want to fund [the development work],” he says. That’s because a smaller potential market for the chemicals makes companies less willing to pour their own resources into developing them.

In the meantime, the partnership is also repurposing a number of existing agricultural insecticides for use in vector control. But Hamon says this is a temporary strategy “because they are out there in agriculture, and in some cases there may already be resistance.”

## Beyond Insecticides

On a hot, clear morning, entomologist Etienne Bilgo sits in the back seat of a car headed toward an IRSS research site in Soumouso, Burkina Faso, where he is attempting to circumvent dependence on chemicals altogether.

“We have to take a little pressure off the insecticides,” says Bilgo, a 29-year-old PhD student under Diabaté. Outside, a green savannah dotted with mango, baobab, and neem trees scrolls by, interrupted here and there by the fields of cotton that have played such a central role in creating that pressure. “We have to find complementary tools. That could be medicine, or hygiene, or reducing breeding sites.”

Tools like these have been crucial in eliminating malaria from wealthier countries around the world. But measures such as draining irrigation ditches, clearing vegetation around houses, and putting screens on windows and eaves must be carefully tailored to each location, and they don’t work everywhere.^17^ Climate change is further complicating the picture by reshuffling the areas in which vectors can thrive.^45^

In Soumouso, Bilgo and Diabaté are preparing to test something different: a transgenic fungus designed by scientists at the University of Maryland, which expresses a toxin produced by scorpions. The fungus, sprayed on mosquitoes, invades the insects’ blood stream and attacks malaria parasites so they cannot be transmitted to people.^46^ ^,^ ^47^ “The best thing about the fungi is they don’t kill the mosquitoes quickly, so they have time to reproduce, and they stay susceptible for many generations,” says Bilgo.

Stepping out of the car, he heads toward a large, low greenhouse-like building whose walls and roof are made of netting. Inside is a replica of a village street, with small mud huts, gardens, and an enclosure full of sleepy cows. Soon, Bilgo will release mosquitoes inside and begin testing the less potent non-transgenic version of the fungus on them as a precursor to importing the transgenic version for tests.

Elsewhere around the world, scientists are working on other new tools to fight malaria, like vaccines^48^ and genetically modified sterile or disease-resistant mosquitoes.^49^ Many are promising. But Strickman, the Gates Foundation program officer, says none provides an immediate escape hatch from the resistance dilemma.

“In the next 20 years, it’s hard to imagine a solution [to malaria] that doesn’t involve insecticides,” he says. Until that solution is found, the actions of farmers, and of the agrochemical companies that supply them with pesticides, will continue to matter greatly in the fight against malaria.
